# Fatal Pyopneumothorax in a COVID-19 Patient

**DOI:** 10.7759/cureus.31866

**Published:** 2022-11-24

**Authors:** Anju Gurung, Dipesh Poudel, Bivek Gurung, Prabhat Rawal, Sunder chapagain

**Affiliations:** 1 Department of Internal Medicine, Nepal Armed Police Force Hospital, Kathmandu, NPL; 2 Department of Anaesthesiology, Nepal Armed Police Force Hospital, Kathmandu, NPL; 3 Department of Internal Medicine, Sherwood Forest Hospitals NHS Foundation/The University of Edinburgh, Nottinghamshire, GBR; 4 Department of Anaesthesiology, Nepal Armed Police Force Hospital, kathmandu, NPL

**Keywords:** bronchopleural fistula, pyopneumothorax, covid 19, thoracostomy tube, chest drain, organized empyema thoracis, multi-drug resistant pathogen

## Abstract

The COVID-19 pandemic has impacted every aspect of our lives since its start in December 2019. Among various COVID-19 complications, pleural complications are also increasingly reported but rarely from Nepal. Here, we presented a case of pyopneumothorax in a 52-year-old male patient referred from another center and admitted to the ICU of Nepal Armed Police Force Hospital with a diagnosis of severe COVID-19 pneumonia in the background of alcohol withdrawal syndrome with delirium tremens and generalized tonic-clonic seizures. He developed a rapid decline in respiratory status with a right-sided pneumothorax and underwent an immediate needle thoracostomy, followed by chest tube insertion. On the sixth day of admission, he had thick yellowish pus in the chest drain (pyopneumothorax), and despite the rigorous efforts in treatment, he died on the 15th day of admission. Though relatively uncommon, clinicians should consider pleural complications like pneumothorax, pleural effusion, pneumomediastinum, and empyema in patients with impaired immune status. In such patients, we should ensure prompt diagnosis with the earliest intervention and rationale use of antibiotics.

## Introduction

Various presentations and complications of COVID-19 have been reported worldwide since it was declared a global pandemic by the World Health Organization (WHO) on March 11, 2020 [1]. The clinical spectrum of COVID-19 varies from asymptomatic (up to 33.3%) or minimal symptoms to clinical illness characterized by acute respiratory failure requiring mechanical ventilation, septic shock, and multiple organ failure [1]. In severe COVID-19 infection, the radiological examination commonly shows bilateral multifocal alveolar opacities that tend to confluence with the total opacification of the lung [1-3]. Though rare, various pleural complications such as pleural effusion, spontaneous pneumothorax, bronchopleural fistula, parapneumonic effusion, and empyema thoracic are reported [2-8]. One such rare complication is pyopneumothorax, defined as the accumulation of air and pus in the pleural cavity. Data regarding pyopneumothorax in COVID-19 patients is limited, with one case report of a child from India [9]. Here, we present a case of pyopneumothorax in a patient diagnosed with COVID-19 pneumonia with severe acute respiratory distress syndrome (ARDS) and the possibility of an immunocompromised state contributing to the fatality despite timely antimicrobial therapy and surgical intervention over 15 days.

## Case presentation

A 52-year-old gentleman, a carpenter by profession, belonging to a lower middle-class economic status, was brought to the COVID intensive care unit (ICU) of our hospital from a district hospital. At presentation, he had tremors, cough, shortness of breath, fever, and altered sensorium. He was treated for pulmonary tuberculosis one year prior to presentation and alcoholic liver disease four years ago. There was no known history of hypertension and diabetes mellitus. He was a chronic smoker (36 years, eight pack years) and a regular consumer of alcohol for 30 years (eye opener for eight years). He had multiple episodes of generalized tonic-clonic seizures (GTCSs) during his two-day stay at the district hospital. Therefore, he was referred to our center for ICU management with the diagnosis of severe COVID pneumonia on the background of alcohol withdrawal syndrome with delirium tremens and GTCSs.

**Figure 1:**
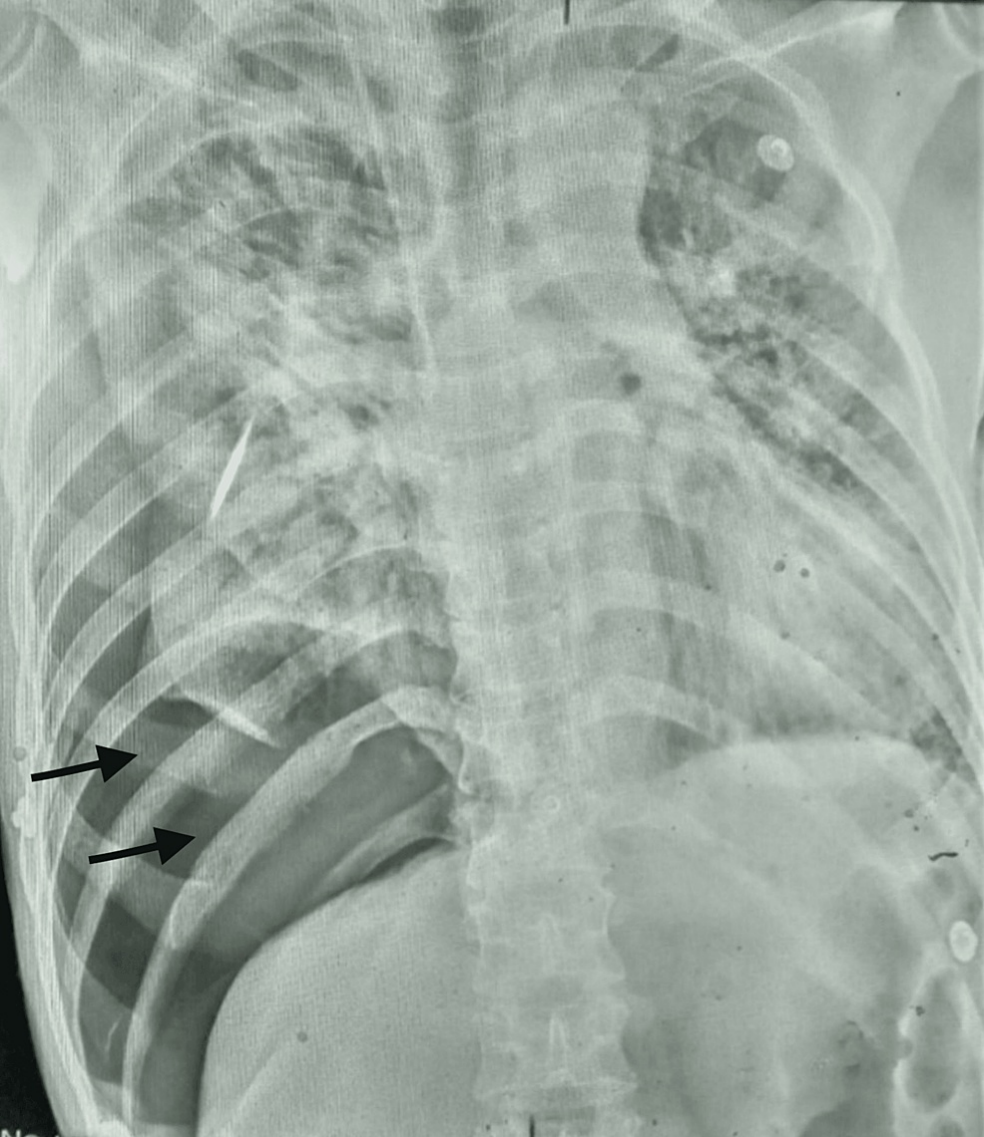
Initial chest X-ray showing right-sided pneumothorax (black arrows).

On initial evaluation, his Glasgow Coma Scale (GCS) was 14/15 (eye-opening 3, verbal response 5, motor response 6). He was tachypneic with a respiratory rate of 32 breaths/min and prominent use of accessory muscles of respiration. He had a blood pressure of 150/110 mm of Hg, pulse rate of 112 beats per minute, and oxygen saturation of 85-90% on 15 L/minute of oxygen via reservoir mask. General random blood sugar was 129 mg/dL, and arterial blood gas analysis showed severe hypoxemia. (APACHE II Score at admission = 14 points). We immediately switched him to bilevel positive airway pressure with the interface of a face mask. However, a gradual decrement in oxygen saturation up to 70% was noticed. A bedside lung ultrasound was performed, which revealed absent lung sliding in the right middle and lower zone and a bar code sign in M-mode suggesting pneumothorax. Chest radiograph showed massive pneumothorax on the right side, with left lower area infiltrates consistent with the diagnosis of COVID-19 pneumonia (Figure 1). Although tension pneumothorax was not our immediate diagnosis, we suspected it was evolving and would ensue soon because of the deterioration in respiratory status. Therefore, we proceeded with immediate needle thoracostomy maintaining standard precautions for asepsis, and the general surgeon on duty was consulted while making necessary preparations for probable chest tube insertion.

**Figure 2:**
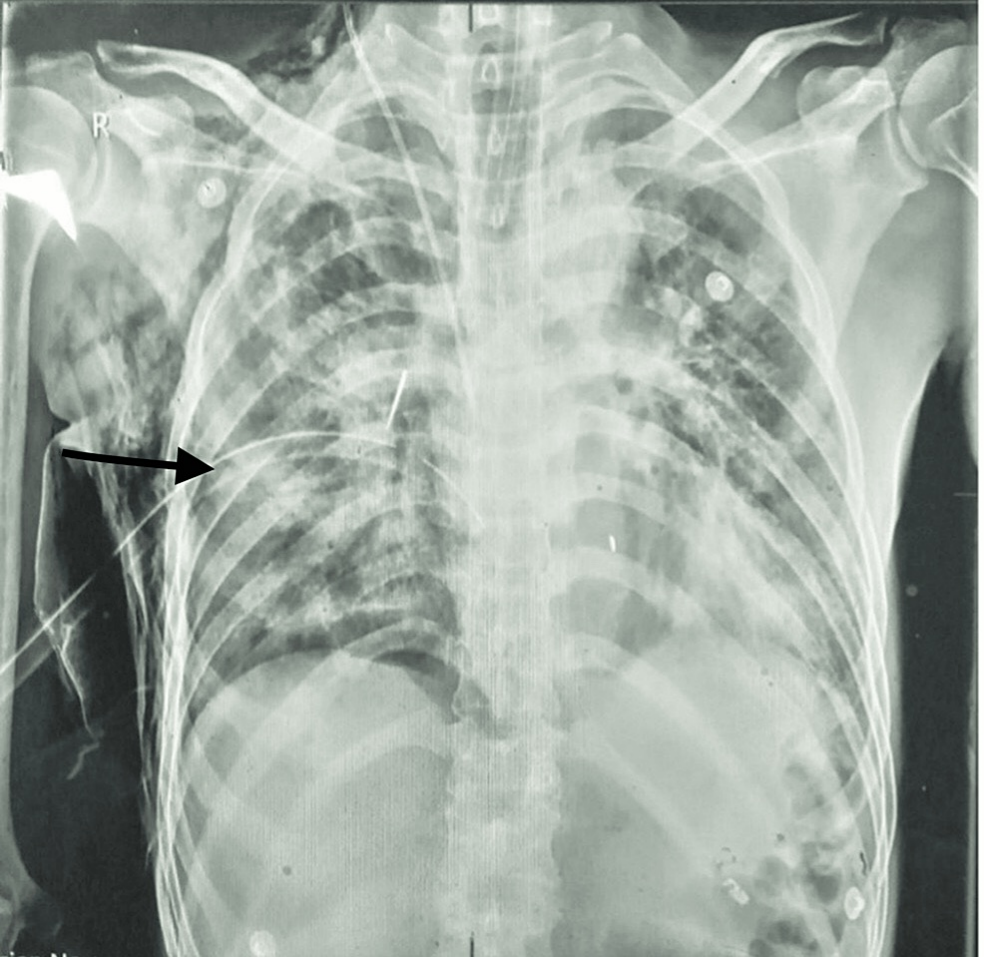
Subsequent chest X-ray after intubation and chest tube placement (black arrow).

Meanwhile, his GCS decreased to 7/15 (E1V2M4), and oxygen saturation dropped below 70%. Hence, rapid sequence intubation was performed. After intubation, oxygen saturation improved to 88-90%. Afterward, bedside tube thoracostomy was performed following an aseptic technique: 24F chest tube drain was inserted on his suitable fifth intercostal space, middle axillary line. Ultrasound-guided central venous catheter was inserted using a modified Seldinger’s technique under aseptic precaution. Chest X-ray was then repeated (Figure 2). We also sent baseline blood investigations.

Blood investigations revealed a white blood cell count of 13,000/cumm with 92% neutrophils and a platelet count of 2,25,000/cumm, hemoglobin of 14 gm/dL, D-dimer of 5.1 mg/L, C-reactive protein of 156 mg/L, peak lactate dehydrogenase of 841 IU/L, urea of 70 mg/dL, creatinine of 1.3 mg/dL, serum sodium of 150 meq/L, serum potassium of 4.2 meq/L, total bilirubin of 0.9 mg/dL, direct bilirubin of 0.3 mg/dL, SGOT (serum glutamic-oxaloacetic transaminase) of 68I U/L, and SGPT (serum glutamic-pyruvic transaminase) of 74 IU/L. Serology for HIV, HBsAg, and HCV was non-reactive (Table 1).

**Table 1:** Sequential blood investigation report WBS, white blood cells; N/L, neutrophil/lymphocytes; PT, prothrombin time; INR, international normalized ratio; APTT, activated partial thromboplastin time; CRP, C-reactive protein; PCT, procalcitonin; LDH, lactate dehydrogenase; IL-6, interleukin 6; RBS, random blood sugar; TB, total bilirubin

Date	08/25	08/27	08/29	09/01	09/03	09/05	09/07	09/08	09/09
Tests	Day 1	Day 3	Day 5	Day 7	Day 9	Day 11	Day 13	Day 14	Day 15
WBC	13,000	14,500	17,600	17,800	20,000	21,500	24,000	25,500	24,650
N/L	92/6	88/7	89/8	92/5	90/5	92/4	93/4	90/6	91/7
Platelets	2,25,000	2,41,000	2,42,000	2,05,000	187,000	196,000	205,000	196,000	191,000
Hemoglobin	14	13.8	14.1	14.1	14	14.1	13.8	13.7	13.6
PT	14	14	17	17	15	17	15	17	16
INR	1.0	1.0	1.3	1.3	1.1	1.3	1.1	1.3	1.2
APTT	45 sec	40 sec	54 sec	60 sec	55 sec	61 sec	65 sec	45 sec	60 sec
CRP	156	176	180	>200	>200	>200	>200	>200	>200
D-dimer	5.1	6.2	4.4	3.9	4.1	3.7	2.2	2.1	2.0
PCT	1.6	1.5	5.9	8.3	5.8	5.0	4.5	2.8	2.0
LDH	841	1050	988	744	821	997	994	851	811
IL-6 (ref<10)			998 pg/mL						
RBS	106	133	200	178	179	205	200	195	208
Urea	87	83	89	70	73	104	140	134	140
Creatinine	1.3	1.2	0.9	1.0	1.4	1.6	1.5	1.4	1.3
Sodium	136	138	134	150	148	145	144	140	141
Potassium	4.7	3.8	3.9	3.7	3.9	3.7	3.1	3.3	3.7
Calcium			8.5	9.2				8.8	
TB	1.0	0.8	0.9	0.8	0.9	0.9	0.8	0.8	0.9

**Figure 3:**
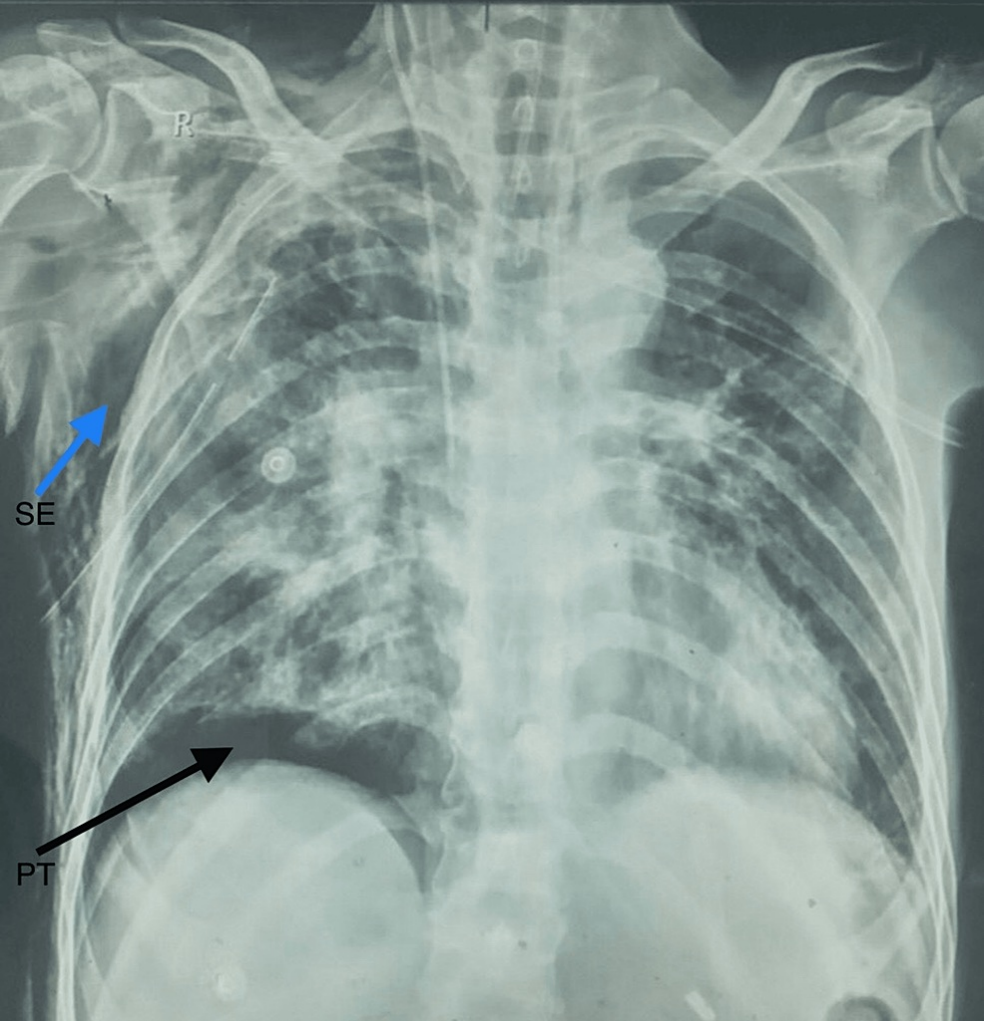
Localized pneumothorax above his right hemidiaphragm (shown by a black arrow) with subcutaneous emphysema (blue arrow). SE, subcutaneous emphysema; PT, pneumothorax

**Figure 4:**
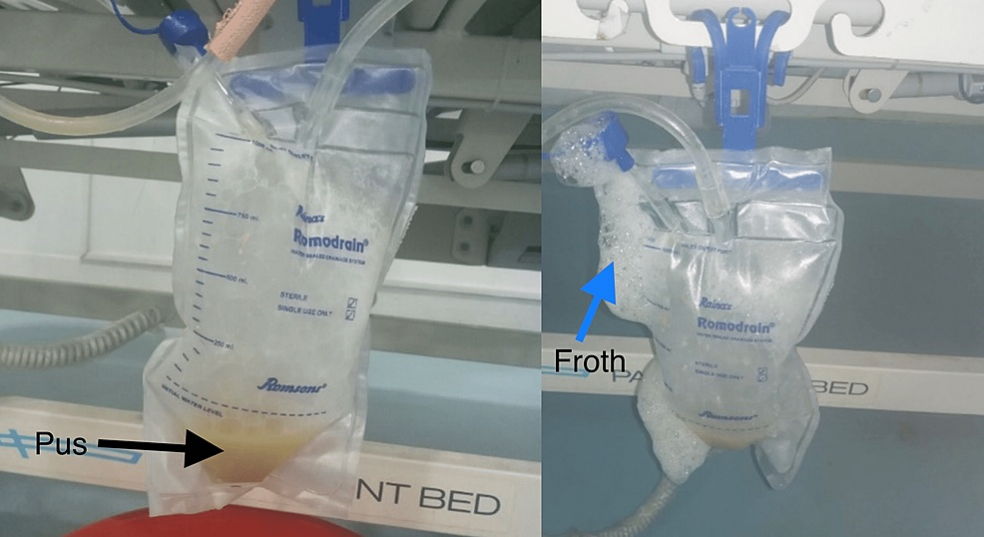
Pus (black arrow) on the chest drain with abundant froth (blue arrow).

There was the full resolution of the pneumothorax on the next day's chest X-ray with minimal water column movement for a few days. However, on the sixth day of his admission, he developed localized pneumothorax above his right hemidiaphragm with subcutaneous emphysema (Figure 3). His vital signs were within the normal range. On that very day, we noticed a thick, yellowish-white, foamy discharge on the chest drain, which was sent for microbiological examination (Figure 4). Multi-drug-resistant (MDR) *Acinetobacter* spp. was isolated after aerobic inoculation for 72 hours from endotracheal tube suction and pus from the pleural cavity. The isolate and minimum inhibitory concentrations (MIC) for various antibiotics were confirmed (Table 2).

**Table 2:** Pleural fluid cytology and culture/sensitivity reports. RME, routine and microscopic examination; WBC, white blood cells

S. No.	Test	Findings
1.	Pleural fluid RME	450 cells /mL, 67% polymorph, and 33% lymphocyte with degenerated WBC
2	Pleural fluid culture and sensitivity	Acinetobacter spp. isolated after 72 hrs of aerobic incubation at 37°C sensitive to polymixin B partially sensitive to colistin resistant to ampicillin, amoxiclav, levofloxacin, ofloxacin, meropenem, erythromycin, cefepime, vancomycin, linezolid, ciprofloxacin, doxycycline

For several days, there was a continuous air leak with frank pus from the chest drain, which was purulent and frothy. Hence, he was clinically diagnosed to have a bronchopleural fistula in conjunction with Pyopneumothorax. However, confirmatory bronchoscopy was not performed. The patient was treated with suitable sensitive antibiotics (polymyxin B, colistin) and antiviral drugs, despite which he eventually succumbed to death on the 15th day of ICU admission.

## Discussion

Pyopneumothorax is the accumulation of viscous, opaque, purulent effusion, and gas in the pleural cavity, which is associated with high morbidity and mortality [10]. Cases of pyopneumothorax in COVID-19 have rarely been reported.

Earlier studies estimated the incidence of pneumothorax in COVID-19 to be approximately 1% in patients requiring hospital admission and 2% in those requiring ICU admission [5]. Furthermore, hydropneumothorax and pyopneumothorax are extremely rare entities. Among pleural complications of COVID-19, few studies have investigated the frequency of parapneumonic effusion and empyema thoracis [3,6]. In a meta-analysis, Saha et al. reviewed 47 observational studies with 4,981 COVID-19 patients. They found a low incidence of 7.3% for parapneumonic pleural effusions, which are more prevalent in the critically ill [6].

It has been found that chronic alcohol consumption suppresses multiple arms of the immune response, leading to an increased risk of infections [11]. Viral infections facilitate secondary bacterial infections by disrupting mucociliary clearance, weakening neutrophil functions, and affecting the functioning of immunological pathways [12]. COVID-19 pathogenesis also involves alveolar rupture and fistulation between the lung parenchyma and the pleura. Aspiration pneumonia could be another factor causing bacterial infection in the lung that spreads to the pleura via a bronchopleural fistula clinically diagnosed in our patient. Since our patient heavily consumed alcohol (CAGE - eye opener) with a history of delirium tremens and several episodes of GTCS, there is a possibility that previous microaspirations have contributed to bacterial infection in the lung. In a patient with a weakened immune system due to chronic alcoholism, bacterial infection can easily superimpose upon the viral infection complicating the pleural effusion into empyema.

Steroids have been proven to be an effective therapeutic option for COVID-19 but may also directly or indirectly predispose to various adverse effects [13]. The immunosuppressive and anti-inflammatory role of steroids benefits patients when used during the cytokine storm of the inflammatory stage of COVID-19 reducing the overall 28-day mortality rate [14]. Our patient was critically ill from COVID-19 pneumonia with severe ARDS, for which he was on high-dose steroids, which could be responsible for the immunosuppressive effect. Besides steroids, thoracostomy could have led to the infection as one of its complications. Although needling could conceivably contribute to pleural infection, there is only anecdotal evidence that this lifesaving procedure results in empyema [15]. Similarly, closed tube thoracostomy is classified as a "clean-contaminated" surgical wound, and empyema thoracis has been reported to be as high as 25% [16]. The presence of bronchopleural fistula further complicated his condition. The global treatment strategy for this complication comprises a three-pronged approach involving the management of the fistula, pleural drainage, and antibiotic therapy [17]. Advanced surgical management of pyopneumothorax, such as video-assisted thoracic surgery (VATS), removes the affected tissue around the lung. However, given the limited array of facilities/equipment, we could not perform VATS.

Pus culture and sensitivity revealed MDR *Acinetobacter baumannii*. *Acinetobacter baumannii* has become a worrying pathogen for nosocomial infection, particularly in ICUs, that displays resistance mechanisms to all existing antibiotic groups and can acquire new determinants of resistance [18,19]. Risk factors independently associated with MDR *A. baumannii* are immunocompromised state, adjunct use of immunosuppressant, respiratory failure at ICU admission, previous antimicrobial therapy, previous sepsis in the ICU, and the number of invasive procedures [20].

Generally, the potential source of infections is contiguous spread from parapneumonic effusion, direct inoculation following the procedure, hematogenous spread of organism, or reactivation of latent infection in pneumothorax or hydrothorax patients. All of these factors, along with the invasive procedures in an immunocompromised state and prolonged hospital stay, resulted in pyopneumothorax.

## Conclusions

To the best of our knowledge, this is the first case of COVID-19 pneumonia with pyopneumothorax reported in Nepal. This case study illustrates that though pleural complications of COVID-19 are uncommon, they can be present in the background of potential risk factors such as immunocompromised status, use of high-dose steroids, chronic alcoholism, and prolonged hospitalization, and, when present, can rapidly lead to mortality, especially when MDR bacteria are involved in the pathogenesis. Clinicians should be vigilant in the appropriateness of immunosuppressants and prompt recognition to prevent the development of fatal sequelae.
